# Estimating the relative load from movement velocity in the seated chest press exercise in older adults

**DOI:** 10.1371/journal.pone.0285386

**Published:** 2023-05-04

**Authors:** Diogo Luís Marques, Henrique Pereira Neiva, Daniel Almeida Marinho, Ivan Miguel Pires, Célia Nunes, Mário Cardoso Marques

**Affiliations:** 1 Department of Sport Sciences, University of Beira Interior, Covilhã, Portugal; 2 Research Center in Sports Sciences, Health Sciences and Human Development (CIDESD), Covilhã, Portugal; 3 Instituto de Telecomunicações, Universidade da Beira Interior, Covilhã, Portugal; 4 Department of Mathematics, University of Beira Interior, Covilhã, Portugal; 5 Centre of Mathematics and Applications, University of Beira Interior, Covilhã, Portugal; University of Urbino Carlo Bo, ITALY

## Abstract

**Aim:**

This study aimed to i) determine the load-velocity relationship in the seated chest press in older adults, ii) compare the magnitude of the relationship between peak and mean velocity with the relative load, and iii) analyze the differences between sexes in movement velocity for each relative load in the chest press.

**Material and methods:**

Thirty-two older adults (17 women and 15 men; 79.6±7.7 years) performed a chest press progressive loading test up to the one-repetition maximum (1RM). The fastest peak and mean velocity reached with each weight were analyzed. Quadratic equations were developed for both sexes and the effectiveness of the regression model was analyzed through a residual analysis. The equations were cross-validated, considering the holdout method. The independent samples t-test analyzed i) the differences in the magnitude of the relationship between peak and mean velocity with the relative load and ii) the differences between sexes in the peak and mean velocity for each relative load.

**Results:**

It was possible to observe very strong quadratic load-velocity relationships in the seated chest press in women (peak velocity: *r*^2^ = 0.97, standard error of the estimate (SEE) = 4.5% 1RM; mean velocity: *r*^2^ = 0.96, SEE = 5.3% 1RM) and men (peak velocity: *r*^2^ = 0.98, SEE = 3.8% 1RM; mean velocity: *r*^2^ = 0.98, SEE = 3.8% 1RM) without differences (*p*>0.05) in the magnitude of the relationship between peak and mean velocity with the relative load. Furthermore, there was no overfitting in the regression models due to the high and positive correlation coefficients (*r* = 0.98–0.99). Finally, men presented higher (*p*<0.001) lifting velocities than women in almost all relative loads, except for 95–100% 1RM (*p*>0.05).

**Conclusion:**

Measuring repetition velocity during the seated chest press is an objective approach to estimating the relative load in older adults. Furthermore, given the velocity differences between older women and men at submaximal loads, it is recommended to use sex-specific equations to estimate and prescribe the relative loads in older adults.

## Introduction

Human aging is a continuous process characterized by progressive loss of muscle mass and reduced ability to produce and apply force in motor tasks such as walking, climbing stairs, and standing up from a chair [1–3]. Therefore, the scientific literature recommends resistance training as a practical and effective approach to counteract the age-related decline in functional capacity and the incidence of falls in older adults [4, 5].

In a geriatric setting, a common practice to determine the resistance training load (intensity) is through the direct measurement of the one-repetition maximum (1RM), which consists of displacing the maximum weight possible in a single lift [6, 7]. Although valid and reliable [8, 9], this is a time-consuming procedure, which makes coaches and clinicians look for more time-efficient methods to estimate the 1RM, such as regression equations based on repetitions-to-failure [7, 10, 11]. Nevertheless, regarding the 1RM procedure, a repetitions-to-failure test can significantly increase the risk of injury, cause high muscle soreness, and delay the recovery time between sessions due to the high physical stress imposed, especially in inexperienced practitioners [7, 9, 10]. In addition, considering the scarcity of equations to estimate the 1RM in older adults, some formulas may not be accurate for individuals of different ages, health conditions, and resistance training backgrounds [7]. Therefore, alternative approaches to estimate the 1RM in older adults are needed, such as measuring repetition velocity at different absolute loads due to the well-known and robust load-velocity relationship [12–14].

Recently, several studies have been performed to analyze the accuracy of repetition velocity to estimate the relative load in individuals with clinical conditions, such as multiple sclerosis [15] and women with previous breast cancer [16], and in older adults without mobility limitations [17, 18]. For example, Marcos-Pardo et al. [17] developed load-velocity equations in the free-weight bench press and 45° inclined leg press with strength-trained older women of ~68 years old. In another study, Marques et al. [18] established sex-specific load-velocity equations in the horizontal leg press with untrained older women and men of ~79 years old. As observed in both studies, the proposed equations for the leg press demonstrated a very high accuracy level (*r*^2^: ~0.91–0.94; standard error of the estimate (SEE): ~5.7% 1RM), suggesting that load-velocity regression equations are accurate to estimate the relative load in geriatric settings [17, 18]. However, Marcos-Pardo et al. [17] did not find the same accuracy level in the free-weight bench press (*r*^2^: 0.83; SEE: 6.10% 1RM). According to the authors, possible reasons for these results could be the lack of a longer familiarization period and the use of free weights instead of resistance machines [17]. Indeed, the use of free weights can increase the variation in the exercise technique because it requires more stabilization and balance than resistance machines [19]. Therefore, future studies should be developed with older women and men proposing load-velocity equations in upper-body exercises performed on resistance machines to analyze their predictive ability to estimate the relative load.

Previous research verified that men achieved higher velocity values than women in almost all relative loads in the horizontal leg press in older adults [18] and the full-back squat in young adults [20]. However, the authors observed that the higher the relative load, the lower the differences between sexes in movement velocity [18, 20]. These data suggest that, regardless of age, women have a greater strength deficit than men. This deficit is the percentage of maximal strength potential not used in a motor task [20, 21]. In addition, other reasons may be associated with larger and stronger type II muscle fiber in the quadriceps in older men than in older women [22, 23], allowing the former to displace submaximal loads at higher velocities. Nevertheless, it remains unclear if these differences between older women and men in movement velocity for the same relative loads also occur in upper-body resistance exercises.

Given the considerations mentioned above, the aims of this study were: i) to analyze the predictive ability of the movement velocity to estimate the relative load in the seated chest press exercise in older women and men, ii) to determine which velocity variable (i.e., peak or mean velocity) presents a stronger relationship with the relative load, and iii) to compare the differences between older women and men in movement velocity for each relative load in the seated chest press. Initially, it was expected to identify a relationship between movement velocity and relative load in the seated chest press in both sexes, as observed in previous studies with younger populations [20, 24, 25]. Consequently, it was expected to observe a stronger relationship between mean velocity and relative load than peak velocity and relative load [16, 26]. Finally, it was hypothesized that men would present higher velocities than women in almost all relative loads in the seated chest press. However, as observed in previous research with younger populations, these differences would decrease as the relative load increases [20, 24, 25].

## Materials and methods

### Participants

Thirty-two older adults (seventeen women and fifteen men) from residential care facilities and day centers volunteered to participate in this study. Inclusion criteria were age ≥ 65, male and female sex, ability to walk and stand up from a chair independently, and willingness to participate in the study and collaborate with the researchers. Exclusion criteria included severe physical dependency (Barthel Index score < 60) and cognitive decline (Mini-Mental State Examination) [MMSE] following the cut-off scores described by Mendes et al. [27]), musculoskeletal injuries in the previous three months, and terminal illness. The clinicians of the centers conducted the initial screening, which included collecting medical information (health problems, Barthel Index questionnaire [28] to assess performance in the activities of daily living), physical performance tests (10-m walking speed [29], five-repetition sit-to-stand [30], handgrip strength [29]), and a cognitive function test (MMSE [31]). Table 1 shows the characteristics of the participants. Regarding the medical information, participants reported an average of 1.0 ± 0.7 health problems with the following frequency: cardiac diseases (22%), hypertension (19%), diabetes (17%), dyslipidemia (11%), respiratory diseases (11%), vision problems (10%), thyroid disorders (9%), digestive system diseases (8%), hearing problems (5%), and osteoarticular problems (5%). The clinicians considered all participants physically and cognitively fit to be included in the study. All participants received detailed information regarding the study procedures and signed a written informed consent. The Ethical Committee of the University of xxx approved this study (code: xxx), which follows the recommendations of the Declaration of Helsinki.

**Table 1:** Participants’ characteristics.

	Women (*n* = 17)	Men (*n* = 15)	Total (*n* = 32)	
Variable	Mean ± SD	Mean ± SD	Mean ± SD	*p-value* ^#^
Age (years)	81.5 ± 7.7	77.5 ± 7.4	79.6 ± 7.7	0.153
Body mass (kg)	65.4 ± 10.5	78.3 ± 15.6	71.5 ± 14.5	0.009
Height (m)	1.49 ± 0.06	1.66 ± 0.08	1.57 ± 0.11	<0.001
BMI (kg/m^2^)	29.5 ± 4.2	28.4 ± 4.9	29.0 ± 4.5	0.175
Barthel Index score	89.1 ± 12.3	90.7 ± 12.9	89.8 ± 12.4	0.731
Education (years)	1.4 ± 2.0	2.7 ± 2.3	2.0 ± 2.2	0.106
MMSE score	20.9 ± 3.6	24.8 ± 4.7	22.7 ± 4.5	0.012
10-m walking test (s)	6.6 ± 1.1	6.9 ± 2.4	6.7 ± 1.8	0.716
5STS test (s)	8.0 ± 1.9	8.7 ± 1.8	8.3 ± 1.9	0.297
HGS absolute (kg)	19.5 ± 4.9	32.9 ± 9.5	25.8 ± 10.0	<0.001
1RM chest press (kg)	27.2 ± 6.4	43.3 ± 11.6	34.7 ± 12.2	<0.001
Relative strength (kg/BM)	0.42 ± 0.11	0.56 ± 0.13	0.49 ± 0.14	0.002

^#^ The independent samples t-test analyzed the differences between older women and men in all variables; SD: standard deviation; 1RM: one-repetition maximum; 5STS: five-repetition sit-to-stand test; BM: body mass; BMI: body mass index; HGS: handgrip strength (the absolute handgrip strength corresponds to the average result of the left and right hands score); MMSE: mini-mental state examination; relative strength = 1RM chest press (kg) / body mass (kg).

### Study design

**Fig 1:**
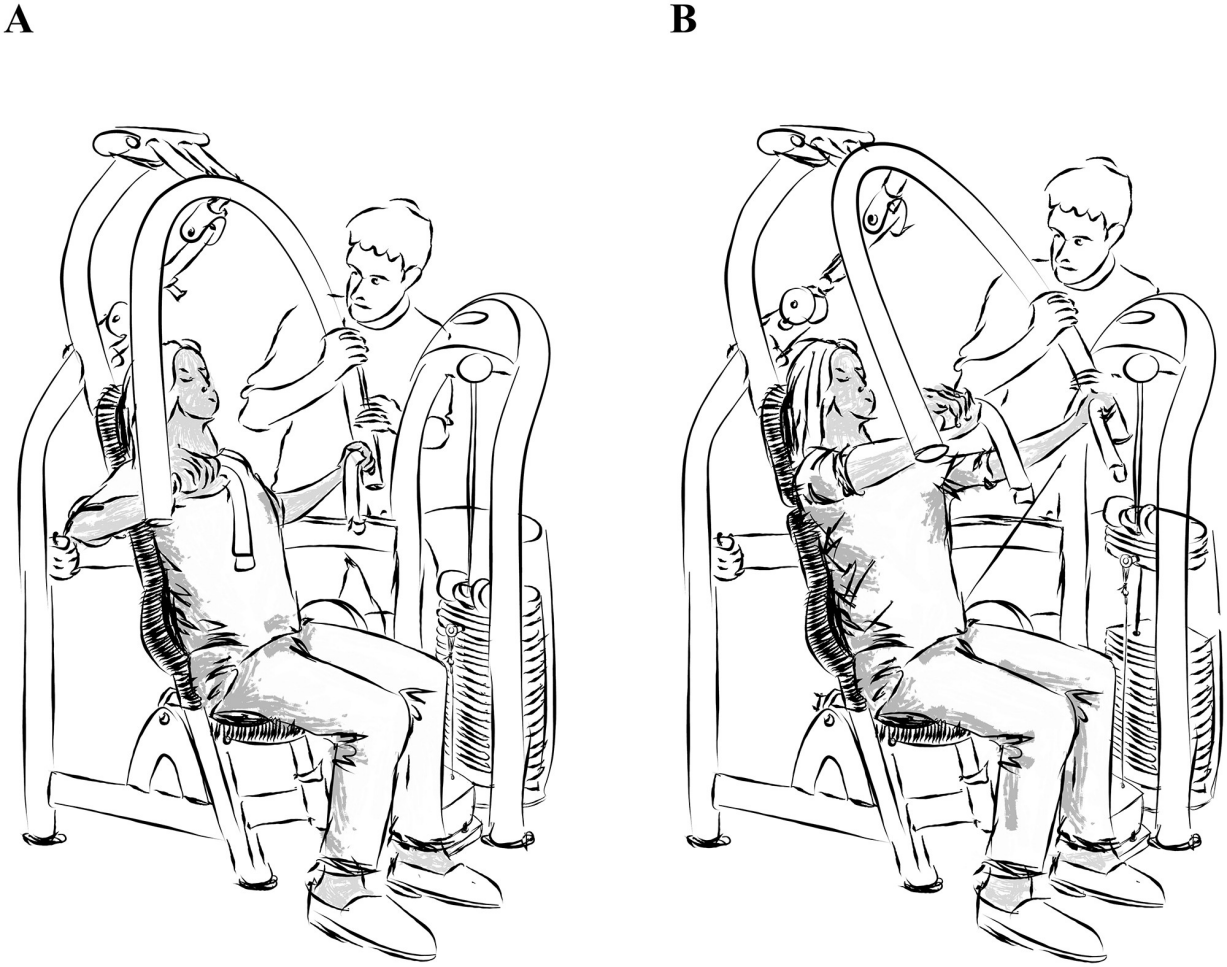
Illustration of the seated chest press machine with the initial (A) and final (B) phases of the movement.

In a cross-sectional study design, untrained older adults went to a fitness health club five times over three weeks from 2 p.m. to 4 p.m. at an ambient temperature of 22–24°C. Four sessions (two each week) were dedicated to familiarization and one to implement the test. In the first week, the participants underwent two sessions with an interval of 48 hours to adapt to the test procedures (focus on exercise technique). Anthropometric measurements were also performed during this period and the correct position was identified for the seated chest press machine (Chest press G3, Matrix, USA) for each participant (Fig 1). In the first session of the second week, all participants performed two sets of five repetitions with 5.7 and 10.2 kg at the intended maximal concentric velocity (focus on movement velocity). After 48 hours of rest, they performed a second session with one set of three repetitions at the intended maximal concentric velocity with 5.7, 10.2, and 14.8 kg and rested three minutes between sets. Finally, in the third week, all participants performed the seated chest press progressive loading test. An experienced researcher and two certified senior coaches’ specialists ensured supervision during all sessions and testing procedures.

### Seated chest press progressive loading test

Initially, the participants warmed up for 10 min by walking on a treadmill at self-selected velocities (2–4 km·h^-1^) or pedaling on a stationary bicycle at self-selected velocities (50–70 rpm) and resistance levels (1–5), followed by 5 min of upper extremity joint mobilization in a seated position (i.e., shoulders and wrists circular rotation back and forth; shoulders, elbows, and wrists flexion and extension). Then, the participants started the test sitting down with the handgrips in the middle of the chest, shoulders abducted, elbows flexed at ~90° (angle adjusted using the machine arms) and grabbing the handles of the machine with a neutral grip (Fig 1). After verbal instruction, the participants fully extended their elbows at the intended maximal concentric velocity. An experienced researcher controlled the concentric phase to prevent the back from losing contact with the back of the machine, particularly with light weights, and the subsequent eccentric phase, placing the hands on the arms of the machine to control the return to the initial position (Fig 1). There was a pause between the eccentric and concentric phases of ~2 s to avoid the rebound effect and provide more consistent measurements [32]. The test warm-up consisted of one set of seven repetitions with 5.7 kg, followed by a set of five repetitions with 10.2 kg. Then the test started with 10.2 kg and progressively increased by ~5 kg using the machine’s weight stacks and extra discs that could be incorporated into it (from 1.1 to 2.3 kg). On average, the first load of the test corresponded to a relative load of 25% 1RM. The participants performed three repetitions with each weight, whenever possible, to enable correct data collection [18, 33]. This method was applied until the participants were able to perform only one correct repetition. In cases where the participants could not perform a single correct repetition, the weight was decreased by 1.1 to 2.3 kg until they could achieve the 1RM. A 3-min rest was provided for three repetitions and a 5-min rest for two repetitions between sets. The average number of sets was 5.4 ± 1.2 for women (total repetitions = 92 [5.4 x 17 participants]) and 7.3 ± 1.9 for men (total repetitions = 110 repetitions [7.3 x 15 participants]).

### Measurement equipment and data collection

The anthropometric measurements included body mass (TANITA BC-601, Japan) and height (Portable stadiometer SECA, Germany). The data was collected using a valid and highly reliable linear velocity transducer (T-Force System, Ergotech, Murcia, Spain) [34, 35], which was coupled to the seated chest press machine (Fig 1), as described in previous research [29]. During each repetition, the T-Force software (v2.36) calculated and displayed in real-time the peak velocity (i.e., the maximum instantaneous velocity value reached during the concentric phase) and the mean velocity (i.e., the average velocity from the start of the concentric phase until the weight stack plate reached the maximum height). However, the mean propulsive velocity (i.e., the portion of the concentric action where the measured acceleration is greater than the acceleration due to gravity (-9.81 m·s^-2^) [36]) was not analyzed because the values were equal to those obtained in the mean velocity, indicating that there was no breaking phase during the concentric action. Therefore, the fastest peak and mean velocities achieved with each weight, including the 5.7 kg weight, were analyzed as participants displaced it with the intended maximal concentric velocity.

### Statistical analysis

The sample size was estimated using the t-test for two independent groups [37]. Twenty-five participants were required to ensure a statistical power of 80%, based on an effect size of 0.60, assuming a standard deviation (SD) of 0.5 (according to the SD of the average number of sets during the free-weight bench press test in Marcos-Pardo et al. [17]), and a significance level of 0.05. Considering a proportion of 53% in the women’s group, 13 women and 12 men were required. Data are presented as mean ± SD and 95% confidence intervals (CI). A regression analysis was carried out to examine the relationship between the movement velocity and relative load in the seated chest press in both sexes. After creating a scatter plot with the independent (peak or mean velocity) and dependent (relative load) variables, the regression model was considered (linear or quadratic) according to the one that provided the best-fit curve to the data. The coefficient of determination (*r*^2^) assessed the predictive ability of the regression equations, the SEE (SD of the residuals) calculated the prediction accuracy, and Pearson’s correlation coefficient (*r*) assessed the relationship between variables. The magnitude of correlation was interpreted as: 0.00–0.10, negligible; 0.10–0.39, weak; 0.40–0.69, moderate; 0.70–0.89, strong; 0.90–1.00, very strong [38]. The verification of the assumptions of normality, independence, and homoscedasticity of the residuals allowed to analyze the effectiveness and adequacy of the regression model [39]. Normality was examined using the histograms, normal P-P plots, and Q-Q plots of the standardized residuals coupled with the Kolmogorov-Smirnov test. Independence was analyzed using the Durbin-Watson test, while the homoscedasticity by inspecting the scatter plots of the standardized residuals against the standardized predicted values. The assumption that there were no extreme values was verified after removing the outliers. The regression equations were cross-validated to test the presence of overfitting. The data was split into two equal-sized subsets, and cross-validation was conducted considering the holdout method.

Individual quadratic regression equations were established for each participant to estimate the peak and mean velocity associated with each relative load. The normality and homogeneity of the data (i.e., estimated peak and mean velocity values for each relative load) were evaluated using the Shapiro-Wilk test and Levene’s test, respectively. The independent samples t-test analyzed i) the differences in the magnitude of the relationship (*r*^*2*^) between peak and mean velocity with the relative load and ii) the differences between sexes in the estimated peak and mean velocity for each relative load. Hedge’s *g* effect size compared the magnitude of sex differences in the estimated peak and mean velocity for each relative load. The effect size (*g*) was interpreted as trivial (0.0–0.2), small (0.2–0.6), moderate (0.6–1.2), large (1.2–2.0), very large (2.0–4.0), and extremely large (> 4.0) [40]. Finally, the between-subject coefficient of variation was calculated to assess the variability of the mean and peak velocity values associated with each relative load, where values below 10% were considered acceptable [16]. The significance level was set at *p* < 0.05. Statistical analyses were performed in Microsoft Office Excel^®^ (Microsoft Inc., Redmond, WA, USA) and SPSS v27 (SPSS Inc., Chicago, IL, USA). The figures were designed in GraphPad Prism v7 (GraphPad Inc., San Diego, CA, USA).

## Results

### Relationship between movement velocity and relative load in both sexes

The quadratic regression model initially provided the best curve fitting on both velocity variables in women and men and was therefore chosen for further analysis. In women, the raw mean velocity data adjusted for linear and quadratic regression produced an *r*^*2*^ of 0.93 and 0.95, respectively, while the raw peak velocity data adjusted for linear and quadratic regression produced an *r*^*2*^ of 0.93 and 0.94, respectively. In men, the raw mean velocity data adjusted for linear and quadratic regression produced an *r*^*2*^ of 0.90 and 0.91, respectively, while the raw peak velocity data adjusted for linear and quadratic regression produced an *r*^*2*^ of 0.90 and 0.91, respectively.

**Fig 2:**
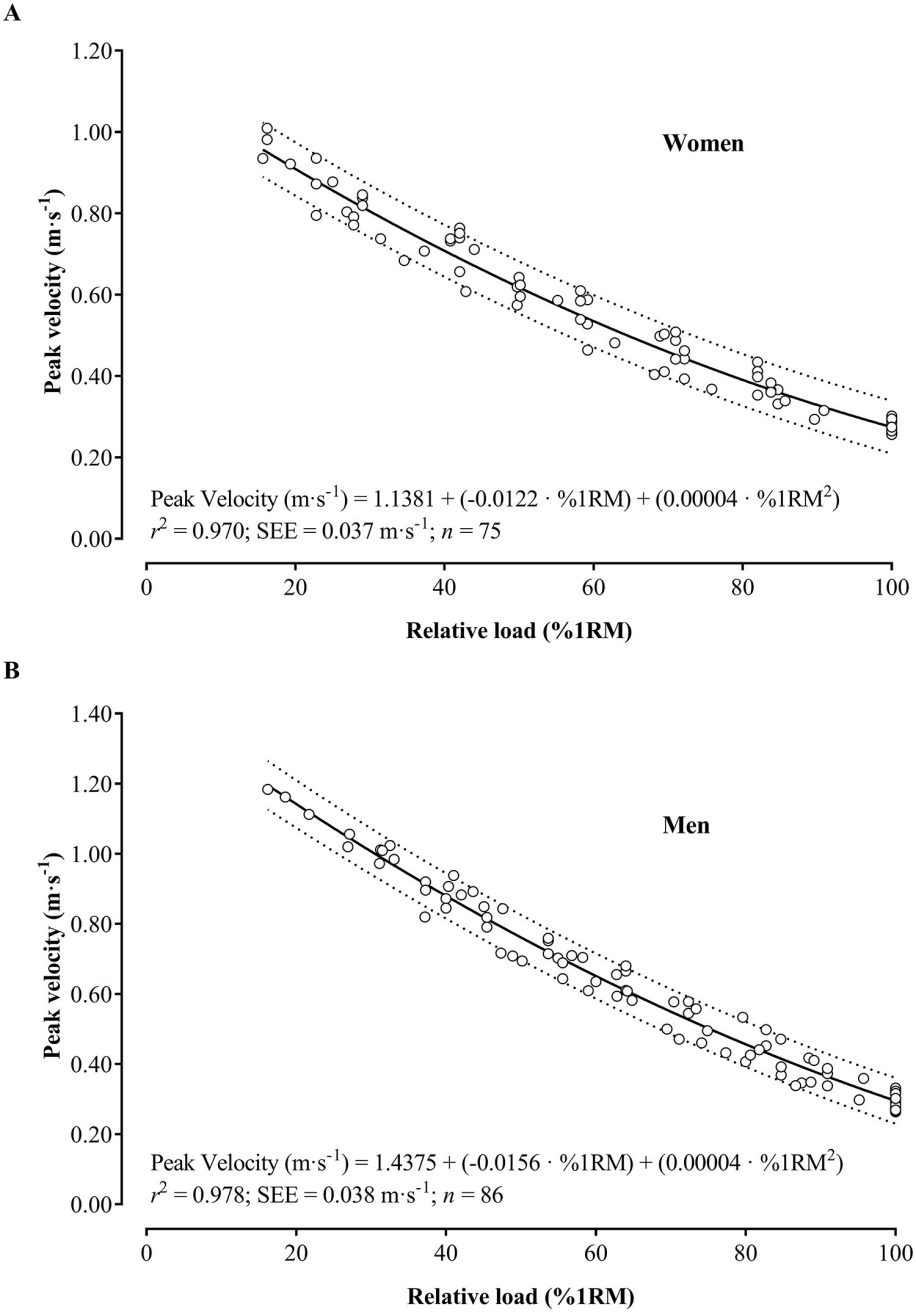
Regression equations to estimate the peak velocity based on the relative load (%1RM) in the seated chest press exercise in older women (A) and men (B). *r*2: coefficient of determination; SEE: standard error of the estimate; n: number of observations; Dotted lines indicate the 95% prediction bands.

**Fig 3:**
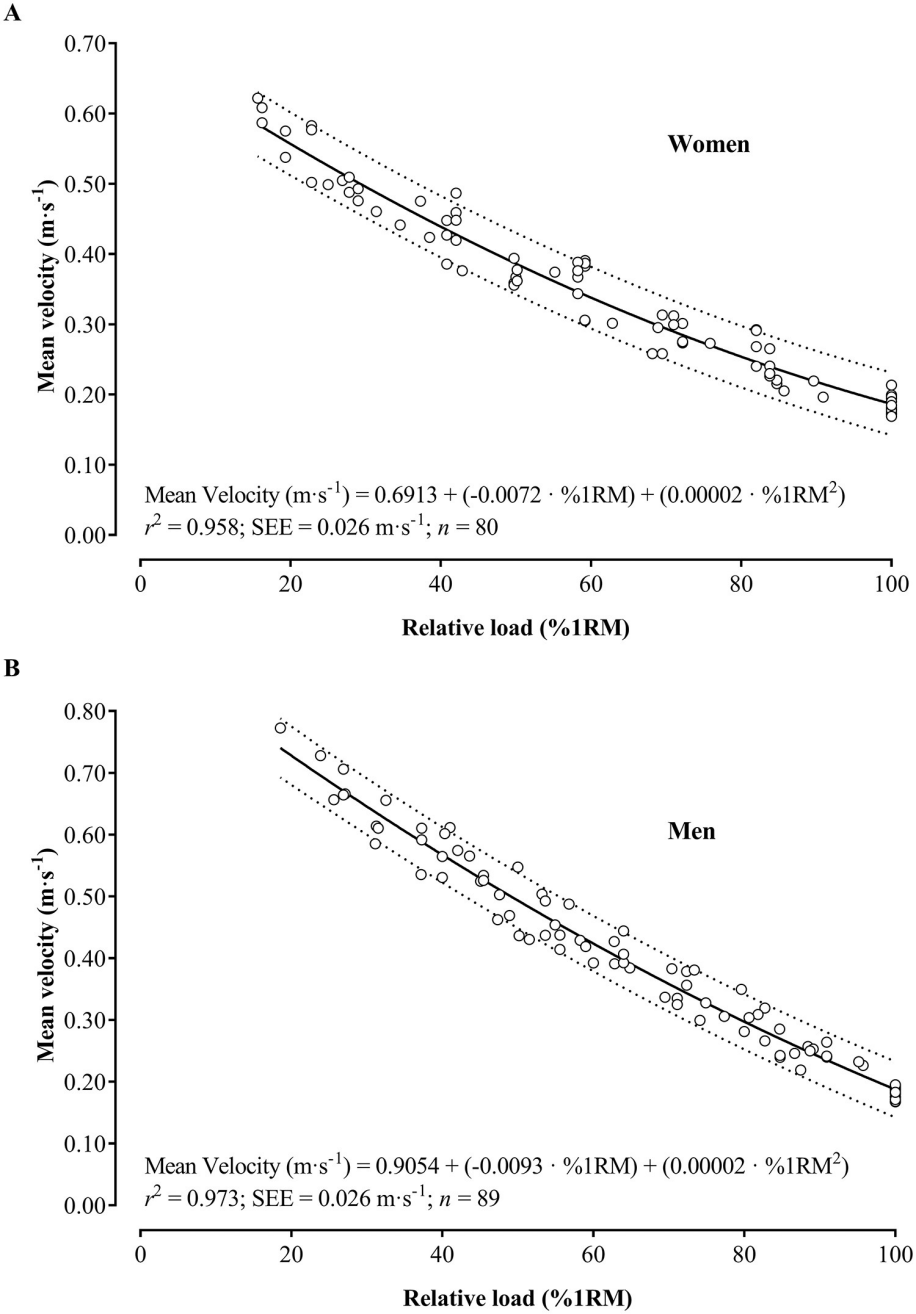
Regression equations to estimate the mean velocity based on the relative load (%1RM) in the seated chest press exercise in older women (A) and men (B). *r*2: coefficient of determination; SEE: standard error of the estimate; n: number of observations; Dotted lines indicate the 95% prediction bands.

While conducting residual analysis in the regression models, the assumption of extreme values was violated due to the presence of large residuals, which consequently led to their removal. Therefore, in women, 17 observations (92 to 75) were removed for the peak velocity analysis (Fig 2A) and 12 (92 to 80) for the mean velocity analysis (Fig 3A). On the other hand, in men, 24 observations (110 to 86) were removed for the peak velocity analysis (Fig 2B) and 21 (110 to 89) for the mean velocity analysis (Fig 3B).

After confirming the assumptions in the quadratic regression models, the results revealed a very strong relationship between the relative load and velocity variables in older women (*r*^*2*^ = 0.96–0.97) and men (*r*^*2*^ = 0.97–98). Figs 2 and 3 show the regression equations to estimate the peak and mean velocity values associated with each relative load, respectively.

Finally, there were no differences (*p* > 0.05) in the magnitude of the relationship between the estimated peak and mean velocity with the relative load in older women (*r*^*2*^ = 0.996 ± 0.003 vs. 0.995 ± 0.005, respectively) and men (*r*^*2*^ = 0.991 ± 0.013 vs. 0.994 ± 0.008, respectively).

### Differences between sexes in the estimated peak and mean velocity for all relative loads

Tables 2 and 3 show the differences between sexes in the estimated peak and mean velocity for each relative load in increments of 5% derived from the individual regression equations, respectively. Men presented higher estimated peak and mean velocity values than women in almost all relative loads (*p* < 0.001), except for 95 and 100% 1RM (*p* > 0.05). There was greater variability at 90–100% 1RM in both velocity variables in women and men and at 20–40% 1RM in men. In women, there was similar variability using the peak and mean velocity (average coefficient of variation: 7.3 and 7.7%, respectively). On the other hand, in men, there was higher variability using peak velocity than mean velocity (average coefficient of variation: 9.2 and 7.5%, respectively).

**Table 2:** Estimated peak velocity for each relative load in the seated chest press exercise in older women and men derived from the individual load-velocity relationships.

Load	Women (m·s^-1^)			Men (m·s^-1^)				Difference	Effect size
(% 1RM)	Mean ± SD	95% CI	CV (%)	Mean ± SD	95% CI	CV (%)	*p-value* ^#^	(m·s^-1^)	Hedge’s *g*
20	0.88 ± 0.08	0.84–0.92	8.8	1.10 ± 0.17	1.01–1.19	15.1	< 0.001	0.22	1.66 (large)
25	0.84 ± 0.07	0.80–0.87	8.4	1.03 ± 0.15	0.95–1.11	14.1	< 0.001	0.20	1.73 (large)
30	0.79 ± 0.06	0.76–0.82	8.2	0.97 ± 0.13	0.90–1.04	13.0	< 0.001	0.18	1.80 (large)
35	0.75 ± 0.06	0.72–0.78	8.0	0.91 ± 0.11	0.85–0.97	12.1	< 0.001	0.17	1.86 (large)
40	0.70 ± 0.06	0.67–0.73	8.0	0.85 ± 0.10	0.80–0.91	11.2	< 0.001	0.15	1.91 (large)
45	0.66 ± 0.05	0.63–0.69	8.0	0.80 ± 0.08	0.75–0.84	10.4	< 0.001	0.14	1.95 (large)
50	0.62 ± 0.05	0.59–0.65	8.0	0.74 ± 0.07	0.70–0.78	9.7	< 0.001	0.12	1.96 (large)
55	0.58 ± 0.05	0.56–0.60	8.1	0.69 ± 0.06	0.66–0.72	9.1	< 0.001	0.11	1.94 (large)
60	0.54 ± 0.04	0.52–0.56	8.1	0.64 ± 0.06	0.61–0.67	8.7	< 0.001	0.10	1.88 (large)
65	0.51 ± 0.04	0.48–0.53	8.2	0.59 ± 0.05	0.56–0.62	8.5	< 0.001	0.08	1.79 (large)
70	0.47 ± 0.04	0.45–0.49	8.2	0.54 ± 0.05	0.52–0.57	8.5	< 0.001	0.07	1.66 (large)
75	0.43 ± 0.04	0.42–0.45	8.3	0.50 ± 0.04	0.47–0.52	8.7	< 0.001	0.06	1.51 (large)
80	0.40 ± 0.03	0.38–0.42	8.4	0.45 ± 0.04	0.43–0.47	9.0	< 0.01	0.05	1.32 (large)
85	0.37 ± 0.03	0.35–0.39	8.7	0.41 ± 0.04	0.39–0.43	9.6	< 0.01	0.04	1.10 (moderate)
90	0.34 ± 0.03	0.32–0.35	9.6	0.37 ± 0.04	0.35–0.39	10.5	0.03	0.03	0.85 (moderate)
95	0.31 ± 0.03	0.29–0.33	11.3	0.33 ± 0.04	0.31–0.35	11.9	0.13	0.02	0.58 (small)
100	0.28 ± 0.04	0.26–0.30	14.2	0.29 ± 0.04	0.27–0.32	14.0	0.38	0.01	0.33 (small)
Average	0.56 ± 0.04	0.54–0.58	7.3	0.66 ± 0.06	0.63–0.69	9.2	< 0.001	0.10	1.97 (large)

^#^ Differences between older women and men in the estimated peak velocity values; SD: standard deviation; 1RM: one-repetition maximum; CI: confidence interval; CV: between-subject coefficient of variation.

**Table 3:** Estimated mean velocity for each relative load in the seated chest press exercise in older women and men derived from the individual load-velocity relationships.

Load	Women (m·s^-1^)			Men (m·s^-1^)				Difference	Effect size
(% 1RM)	Mean ± SD	95% CI	CV (%)	Mean ± SD	95% CI	CV (%)	*p-value* ^#^	(m·s^-1^)	Hedge’s *g*
20	0.55 ± 0.05	0.52–0.57	9.1	0.72 ± 0.09	0.67–0.77	11.9	< 0.001	0.17	2.49 (very large)
25	0.52 ± 0.04	0.50–0.54	8.5	0.68 ± 0.08	0.64–0.72	11.3	< 0.001	0.16	2.61 (very large)
30	0.49 ± 0.04	0.47–0.51	8.0	0.64 ± 0.07	0.60–0.68	10.7	< 0.001	0.15	2.71 (very large)
35	0.46 ± 0.04	0.45–0.48	7.8	0.60 ± 0.06	0.57–0.63	10.0	< 0.001	0.14	2.79 (very large)
40	0.44 ± 0.03	0.42–0.45	7.7	0.56 ± 0.05	0.53–0.59	9.4	< 0.001	0.13	2.85 (very large)
45	0.41 ± 0.03	0.39–0.42	7.9	0.52 ± 0.05	0.50–0.55	8.8	< 0.001	0.11	2.87 (very large)
50	0.38 ± 0.03	0.37–0.40	8.1	0.49 ± 0.04	0.47–0.51	8.2	< 0.001	0.10	2.86 (very large)
55	0.36 ± 0.03	0.35–0.37	8.4	0.45 ± 0.03	0.43–0.47	7.6	< 0.001	0.09	2.79 (very large)
60	0.34 ± 0.03	0.32–0.35	8.7	0.42 ± 0.03	0.40–0.43	7.1	< 0.001	0.08	2.69 (very large)
65	0.31 ± 0.03	0.30–0.33	9.1	0.38 ± 0.03	0.37–0.40	6.6	< 0.001	0.07	2.53 (very large)
70	0.29 ± 0.03	0.28–0.30	9.4	0.35 ± 0.02	0.34–0.36	6.2	< 0.001	0.06	2.33 (very large)
75	0.27 ± 0.03	0.26–0.28	9.7	0.32 ± 0.02	0.31–0.33	6.2	< 0.001	0.05	2.06 (very large)
80	0.25 ± 0.02	0.24–0.26	10.0	0.29 ± 0.02	0.28–0.30	6.5	< 0.001	0.04	1.72 (large)
85	0.23 ± 0.02	0.22–0.24	10.5	0.26 ± 0.02	0.25–0.27	7.5	< 0.001	0.03	1.32 (large)
90	0.21 ± 0.02	0.20–0.22	11.4	0.23 ± 0.02	0.22–0.24	9.3	0.02	0.02	0.87 (moderate)
95	0.19 ± 0.03	0.18–0.21	13.1	0.20 ± 0.02	0.19–0.22	12.1	0.24	0.01	0.43 (small)
100	0.18 ± 0.03	0.16–0.19	16.0	0.18 ± 0.03	0.16–0.19	16.2	0.86	0.002	0.07 (trivial)
Average	0.35 ± 0.03	0.33–0.36	7.7	0.43 ± 0.03	0.41–0.45	7.5	< 0.001	0.08	2.80 (very large)

^#^ Differences between older women and men in the estimated mean velocity values; SD: standard deviation; 1RM: one-repetition maximum; CI: confidence interval; CV: between-subject coefficient of variation.

### Estimating the relative load from the peak and mean velocity

The following sex-specific equations were obtained to estimate the relative load from peak velocity:

$$\text{Women}:\text{Load}\;\left(\%\;1\text{RM}\right)=149.37+\left(-205.01\;\cdot \;\text{Peak}\;\text{Velocity}\right)+\left(70.871\;\cdot {\;\text{Peak}\;\text{Velocity}}^{2}\right)$$

$$\left(r=-0.986;\;r^{2}=0.972;\;\text{SEE}=4.547\%\;1\text{RM}\right)$$

$$\text{Men}:\text{Load}\;\left(\%\;1\text{RM}\right)=136.09+\left(-136.88\;\cdot \;\text{Peak}\;\text{Velocity}\right)+\left(31.699\;\cdot {\;\text{Peak}\;\text{Velocity}}^{2}\right)$$

$$\left(r=-0.988;\;r^{2}=0.976;\;\text{SEE}=3.808\%\;1\text{RM}\right)$$

Additionally, to estimate the relative load from mean velocity, the sex-specific equations obtained were the following:

$$\text{Women}:\text{Load}\;\left(\%1\text{RM}\right)=156.36+\left(-350.98\;\cdot \;\text{Mean}\;\text{Velocity}\right)+\left(196.04\;\cdot {\;\text{Mean}\;\text{Velocity}}^{2}\right)$$

$$\left(r=-0.988;\;r^{2}=0.960;\;\text{SEE}=5.319\%\;1\text{RM}\right)$$

$$\text{Men}:\;\text{Load}\left(\%1\text{RM}\right)=136.58+\left(-214.50\;\cdot \;\text{Mean}\;\text{Velocity}\right)+\left(79.025\;\cdot {\;\text{Mean}\;\text{Velocity}}^{2}\right)$$

$$\left(r=-0.988;\;r^{2}=0.975;\;\text{SEE}=3.809\%\;1\text{RM}\right)$$

Table 4 presents the cross-validation procedure for quadratic equations using the peak and mean velocity. As can be seen through the high correlation coefficient values (*r* = 0.977–0.990) and the trivial differences between both subsets, the data suggest no overfitting presence.

**Table 4:** Cross-validation using the holdout method.

	Relative load (% 1RM)	Testing set^#^	Training set^#^
Peak velocity	Women	0.984	0.988
	Men	0.988	0.990
Mean velocity	Women	0.977	0.981
	Men	0.990	0.985

^#^ Pearson correlation coefficient between predicted and observed values.

## Discussion

This study analyzed the predictive ability of the movement velocity to estimate the relative load in the seated chest press exercise in older women and men. The main findings can be synthesized as follows: i) there was a very strong quadratic load-velocity relationship in the seated chest press in older women and men; ii) both velocity variables presented a very strong relationship with the relative load, without differences between them; iii) men presented higher velocities than women in almost all relative loads, except for 95 and 100% 1RM. Therefore, these results indicate that measuring intended maximal concentric velocity during the seated chest press is a practical approach to accurately determine the relative load in older women and men.

In previous research, Marcos-Pardo et al. [17] analyzed the load-velocity relationship in the free-weight bench press in strength-trained older women. The equation proposed in that study presented lower *r*^2^ (0.83) and higher SEE (6.10% 1RM) values than those observed in the present study. Two possible explanations for these differences can be considered. One possibility could be that the use of a resistance machine instead of free weights allowed a linear cable movement, which increased its stabilization and avoided oscillations adding noise to data collection. Another possibility could be that the statistical approach used in the present study included testing the assumptions of the regression models through residual analysis. This is a procedure suggested by the specialized literature [39], which increased its goodness of fit. Therefore, the present results suggest that the accuracy of the load-velocity regression equations in older adults can be high when using resistance machines and adequate statistical procedures, namely residual analysis. Nevertheless, future research with older women and men should compare the predictive ability of the load-velocity relationship of the seated chest press vs. free-weight bench press using similar statistical methods for plausible comparisons between both forms of exercise.

The results of this study showed no differences in the magnitude of the relationship between peak and mean velocity with the relative load, suggesting that both variables seem appropriate to determine the relative load in the seated chest press exercise in older adults. Nevertheless, it is important to point out that peak velocity is generally considered to be more suitable for measuring ballistic movements (e.g., throws and jumps), as it captures the fastest point during the concentric portion [41–45]. On the other hand, as mean velocity captures the entire concentric phase of a movement, it may be better suited for performing traditional resistance exercises (e.g., leg press and chest press), as it can be more stable than peak velocity [16]. Importantly, these results showed that older men presented, on average, higher between-subject variability in the seated chest press progressive loading test when using peak velocity instead of mean velocity. Therefore, as the previous literature suggested, mean velocity might be more appropriate for measuring and monitoring the relative load in resistance machine-based exercises [16].

The current study demonstrated that older men presented higher movement velocity values than older women in almost all relative loads in the seated chest press, except for 95 and 100% 1RM. Previous research with physically active young adults corroborates these results demonstrating that men present higher lifting velocity values than women in almost all relative loads in the bench press, except for heavier loads (~80–100% 1RM) [20, 24, 25]. Therefore, these results suggest that the differences between sexes in movement velocity decrease as the relative loads increase in young and older adults. One of the reasons may be associated with a greater strength deficit in women [20, 21], which prevents them from moving light to moderate loads at higher velocities than men. However, these differences are attenuated as the relative loads approach the maximum because the velocities are naturally lower, which means more dependency on the load than on the individual’s capacities. Therefore, given the differences, these data reinforce the pertinence of modeling sex-specific load-velocity regression equations for young [20, 24, 25] and older adults in the chest press.

In the present study, older women reached, on average, lower mean velocities than those observed in a previous study with strength-trained older women (~0.07 m·s^-1^ difference) [17]. These differences are probably related to age (~11 years difference), training experience (trained vs. untrained), and form of exercise (free-weight vs. resistance machine). On the other hand, the male participants of this study reached, on average, similar mean velocities (~0.01 m·s^-1^ difference) than strength-trained older women for the same relative loads [17]. Although in the leg press, similar findings were observed in research with older adults [18], suggesting that load-velocity regression equations should be established based on age, sex, training experience, and form of exercise. Additionally, it is essential to note that research with female breast cancer survivors suggested that load-velocity regression equations should also be established based on the type of pathology [16].

In line with previous research [17], these results showed that older adults achieve lower lifting velocities than trained young adults with the same relative loads in resistance exercises that recruit the chest muscles (e.g., bench press) [12, 20, 24, 25]. Furthermore, when looking at differences in velocity values at 5% increments of the relative load, both older women and men present a narrower range of mean velocities (~0.03 m·s^-1^) than physically active young adult women (~0.07 m·s^-1^) and men (~0.08 m·s^-1^) [12, 20, 24, 25]. These data may reflect an impaired force-generating capacity in older adults, attributed to the reduced type II muscle fiber size [22, 46]. Therefore, to mitigate the age-related loss of muscle fiber size and cross-sectional area, older adults should be encouraged to work out against external resistances to increase type II fiber size and improve the ability to apply force rapidly [47–49].

The estimated velocity values presented in the current study for each relative load in older women and men can have high practical relevance in clinical, training, and research contexts. The first point is that the velocity values can be used as a reference for prescribing relative loads during a resistance training program, assuming that the repetition velocity will be recorded in all sessions. This way of prescribing the relative load has the advantage of knowing in real-time whether the participant is training according to the programmed relative load and adjusting the absolute load if large deviations from the target velocity are observed [12–14, 50, 51]. Indeed, prescribing the relative loads based on specific velocities instead of percentages of 1RM has increased in the last few years in research and training settings with younger populations [14, 50–53]. However, these procedures have never been performed in geriatric settings, which means that future studies using a velocity-monitored resistance training approach should be conducted to test its feasibility in older adults. A second point worth mentioning is that by considering velocity differences between each 5% 1RM, the data will allow coaches and researchers to quantify strength adaptations after a few training sessions, a pre-post training intervention, or a detraining period [12]. Therefore, knowing these values will be essential to obtain information about the changes in the individual load-velocity profile after periods of training, detraining, and retraining.

This study presents several limitations that need to be addressed. First, a larger sample size would allow generalizing the proposed sex-specific regression equations to other older individuals. Second, although there were no significant differences between older women and men in age (a 4-year difference), recruiting a sample with a minimal age difference would perhaps allow for more accurate results regarding the differences between sexes in movement velocity for each relative load. Third, comparing the velocities obtained in the resistance machine versus a different one (e.g., with a different number and distribution of levers) would be necessary to understand the transferability of the results and the accuracy of the regression equations when using different machines. Finally, comparing the load-velocity relationship in the horizontal leg press and seated chest press in both sexes would allow a direct analysis of the differences in the movement velocity in all relative loads in both exercises.

## Conclusions

The current research demonstrates that it is possible to accurately determine the load-velocity relationship in the seated chest press exercise in older adults, a widely used exercise in geriatric research and fitness facilities. Therefore, the provided velocity values associated with each relative load and the proposed sex-specific equations may be useful for clinicians, coaches, and researchers to implement velocity-monitored resistance training in older adults and analyze the changes in the load-velocity profile in the seated chest press throughout its course.

## Supporting information

S1 Data (XLSX)
